# The degree of mitochondrial DNA methylation in tumor models of glioblastoma and osteosarcoma

**DOI:** 10.1186/s13148-018-0590-0

**Published:** 2018-12-17

**Authors:** Xin Sun, Vijesh Vaghjiani, W. Samantha N. Jayasekara, Jason E. Cain, Justin C. St. John

**Affiliations:** 1 Mitochondrial Genetics Group, Hudson Institute of Medical Research, 27-31 Wright Street, Clayton, VIC 3168 Australia; 2 Department of Molecular and Translational Sciences, Faculty of Medicine, Nursing and Health Sciences, Monash University, 27-31 Wright Street, Clayton, VIC 3168 Australia; 3 Centre for Cancer Research, Hudson Institute of Medical Research, 27-31 Wright Street, Clayton, VIC 3168 Australia

**Keywords:** Mitochondrial DNA, mtDNA copy number, mtDNA haplotype, DNA methylation, Glioblastoma multiforme, Osteosarcoma

## Abstract

**Background:**

Different cell types possess different copies of mtDNA to support their specific requirements for cellular metabolism. Cell-specific mtDNA copy numbers are established through cell-specific mtDNA replication during cell differentiation. However, cancer cells are trapped in a “pseudo-differentiated” state as they fail to expand mtDNA copy number. Global DNA methylation can regulate this process, as induced DNA demethylation promotes differentiation of cancer cells and expansion of mtDNA copy number.

**Results:**

To determine the role that mtDNA methylation plays in regulating mtDNA replication during tumorigenesis, we have characterized the patterns of mtDNA methylation using glioblastoma and osteosarcoma tumor models that have different combinations of mtDNA genotypes and copy number against common nuclear genome backgrounds at different stages of tumor progression. To ensure the reliability of the findings, we have applied a robust experimental pipeline including three approaches, namely whole-mtDNA bisulfite-sequencing with mtDNA-genotype-specific analysis, pyrosequencing, and methylated immunoprecipitation against 5mC and 5hmC. We have determined genotype-specific methylation profiles, which were modulated through tumor progression. Moreover, a strong influence from the nuclear genome was also observed on mtDNA methylation patterns using the same mtDNA genotype under different nuclear genomes. Furthermore, the numbers of mtDNA copy in tumor-initiating cells affected mtDNA methylation levels in late-stage tumors.

**Conclusions:**

Our findings highlight the influences that the nuclear and mitochondrial genomes have in setting mtDNA methylation patterns to regulate mtDNA copy number in tumorigenesis. They have important implications for assessing global DNA methylation patterns in tumorigenesis and the availability of mtDNA template for mtDNA replication.

**Electronic supplementary material:**

The online version of this article (10.1186/s13148-018-0590-0) contains supplementary material, which is available to authorized users.

## Introduction

In human cells, mitochondria possess their own genome, mitochondrial DNA (mtDNA). mtDNA is organized as a circular, double-stranded structure [1], which is maternally-only inherited. The human mitochondrial genome is 16,569 bp in size and is present in multiple copies per cell [1]. It is essential for cellular function as it encodes 13 subunits of the electron transfer chain (ETC) complexes, where oxidative phosphorylation (OXPHOS) takes place, the major pathway for generating cellular energy. mtDNA also encodes 22 transfer RNAs (tRNAs) and 2 ribosomal RNAs (rRNAs). In contrast to the nuclear genome, the mitochondrial genome has only two non-coding regions. The major non-coding region, the D-loop, contains the transcription promoter sites for the heavy (HSP1/2) and light strands (LSP), and the initiation site for heavy strand replication (O_H_). As a result, this region is the site of interaction for the nuclear-encoded transcription and replication factors that translocate to the mitochondrion to mediate mitochondrial genomic activities [1]. The other non-coding region is located two-thirds downstream, and contains the initiation site for light strand replication (O_L_) [1].

mtDNA copy number varies according to cell type. This is established during early development when mtDNA replication is strictly regulated in order that a differentiated cell is able to acquire sufficient copies of mtDNA to meet its specific energy requirements [2, 3]. Indeed, successful differentiation is subject to a naïve cell establishing the mtDNA set point, which is defined as the lowest number of mtDNA copies required to be present (~ 200 copies) to act as initiating templates for replication as the process of differentiation ensues [2–6]. However, mtDNA replication appears to be blocked in cancer cells as they undergo differentiation [6]. As a result, a number of cancer cell types maintain low numbers of mtDNA copy as they primarily rely on aerobic glycolysis for energy production, which promotes cellular proliferation at the expense of cell differentiation [4]. Indeed, cancer cells are trapped in a “pseudo-differentiated” state whereby they are unable to maintain or reinforce the mtDNA set point and, therefore, failed to expand mtDNA copy number [7, 8].

The existence of mtDNA methylation has been debated in recent years with a number of datasets either supporting or rejecting the presence of methylated sites in the mitochondrial genome [9–12]. Some of the debates have been centered around the technical limitations in determining the extent of mtDNA methylation. In particular, it has been reported that the circular and supercoiling structure of the mitochondrial genome could interfere with the bisulfite treatment and cause overestimation of the levels of mtDNA methylation [13] and that the mitochondrial genome requires linearization prior to bisulfite conversion or immunoprecipitation of methylated DNA [14]. However, with increasing attention associated with mtDNA methylation, it has become apparent that DNA methylation could take place in the mitochondrial genome, especially in the main non-coding region [14–16], which contains the regulatory regions O_H_, HSP1/2, and LSP. This is further supported by the finding that DNA methyltransferases, namely the DNMT enzymes, and DNA demethylation enzymes, the ten-eleven translocation methylcytosine dioxygenases (TET), have been found in mitochondria [17, 18]. The DNMT enzymes methylate cytosine into 5-methylcytosine (5mC), whereas the TET enzymes promote DNA demethylation by oxidizing 5mC to 5-hydroxymethylcytosine (5hmC) [17, 18]. This is also consistent with our previous finding that the DNA demethylation agents vitamin C (VitC), the activator of TET, and 5-Azacytidine (5Aza), the inhibitor of DNMT, were able to significantly reduce levels of mtDNA methylation [16]. Consequently, the use of DNA demethylation agents could promote tumor cells to complete differentiation and expand mtDNA copy number, which is mediated by the levels of DNA methylation at exon 2 of *POLG*, the gene encoding the mtDNA-specific polymerase gamma, and exon 8 of *TOP1MT*, the gene encoding the mtDNA-specific topoisomerase [4, 16, 19]. Furthermore, as mtDNA appears to synergistically undergo DNA demethylation [16], it is likely that the DNA demethylation of mtDNA would provide extra template to promote mtDNA replication. This indicates an extra epigenetic layer in the regulation of mtDNA replication.

Importantly, another feature of mtDNA is its susceptibility to variants [20]. mtDNA haplotypes, defined by the phylogenetic origins of their maternally inherited lineages [21], have been associated with a range of human diseases such as cancer [22], diabetes [23], Alzheimer’s [24] and Parkinson’s [25], and infertility [26, 27]. Different mtDNA genotypes are able to induce differential gene expression patterns of the same nuclear genome in stem cell [28] and tumor [5] models. Therefore, it is of great importance to take mtDNA genotypes into consideration when investigating mtDNA methylation. For instance, to determine mtDNA methylation levels using whole-genome bisulfite-sequencing technology, non-specific mapping to the published human reference genome, such as the hg38 Human Genome Assembly, could potentially produce over- or underestimation of mtDNA methylation levels.

In order to determine the degree that mtDNA methylation regulates mtDNA replication during tumorigenesis, we have characterized the patterns of mtDNA methylation using tumor models of glioblastoma and osteosarcoma that have different combinations of mtDNA genotypes and copy number against the same nuclear genome background at different stages of tumor progression. The tumor models, which we had previously generated, were derived from the 143B cell line and a mtDNA-depleted 143B cell line repopulated with donor mtDNA from HSR-GBM1 cells and human neural stem cells (hNSCs) [5]; and from HSR-GBM cells depleted of their mtDNA content to varying degrees [6]. We have applied three types of approaches including whole-mitochondrial genome bisulfite-sequencing with mtDNA-genotype-specific analysis, methylated immunoprecipitation (MeDIP), and pyrosequencing. The findings provide novel insights into mtDNA methylation and its role in the epigenetic regulation of mtDNA copy number in tumorigenesis.

## Results

### Characterization of genotype-specific mtDNA methylation through bisulfite sequencing

By isolating mitochondria and purifying mtDNA from early (~ 50 mm^3^) and late (~ 800 mm^3^) stage tumors that possessed the same chromosomal backgrounds but different mtDNA genotypes from osteosarcoma (143B) cells, glioblastoma multiforme (GBM) cells, and hNSC, namely 143B^143B^, 143B^GBM^, and 143B^NSC^ tumors, and performing whole-genome-bisulfite sequencing, we were able to achieve a coverage of more than 500-fold on average across the mitochondrial genome among the tumors. This is deemed to be excellent in terms of mtDNA bisulfite sequencing analysis (Additional file 1) and provides sufficient depth to identify the overall levels of mtDNA methylation at individual sites [13].

In order to determine the specific mtDNA methylation profiles for each genotype, we first generated long PCR products as linearized control samples for each mtDNA genotype to ensure the correct context for each comparison. Each long PCR control consisted of equal concentrations of two overlapping sequences (~ 8500 kb) that span the whole mitochondrial genome. The positive control samples were treated with *M.SssI* CpG methyltransferase to identify the methylated bases for each genotype and the levels of methylation at each site. The negative controls were not treated with the enzyme and would act as the baseline, indicative of non-mtDNA methylation. Notably, we have applied mtDNA genotype-specific mapping to each cohort of bisulfite sequencing outputs using our previously published mtDNA sequences for each genotype [5]. The complete mtDNA sequences for the 143B^143B^, 143B^GBM^ and 143B^NSC^ genotypes are available in GenBank nucleotide core databases under accession numbers KT946592, KT946593, and KT946594, respectively [5]. In the published human mitochondrial genome (hg38), there are a total of 870 CpG predicted sites on both strands of the mitochondrial genome [14], which is the same for the 143B^NSC^ genotype (Additional file 2). The differences in CpG profiles among the mitochondrial genomes of the 143B^143B^, 143B^GBM^, and 143B^NSC^ tumors are evident at nt 9053–9054 and nt 11911–11912, which are only present in the 143B^143B^ and 143B^GBM^ genomes (Additional file 2). CpG sites that were validated by the long PCR samples were kept for further analysis. These had a minimum of 10 reads at each site across all control samples. In total, there were 810 CpG sites from both strands validated for further comparisons (Additional file 3). The full potential of DNA methylation at each CpG site determined by the positive and negative linearized control samples demonstrated the feasibility of mtDNA to undergo methylation and provided the baseline for genotypic data normalization in subsequent analyses (Additional file 3).

**Fig. 1:**
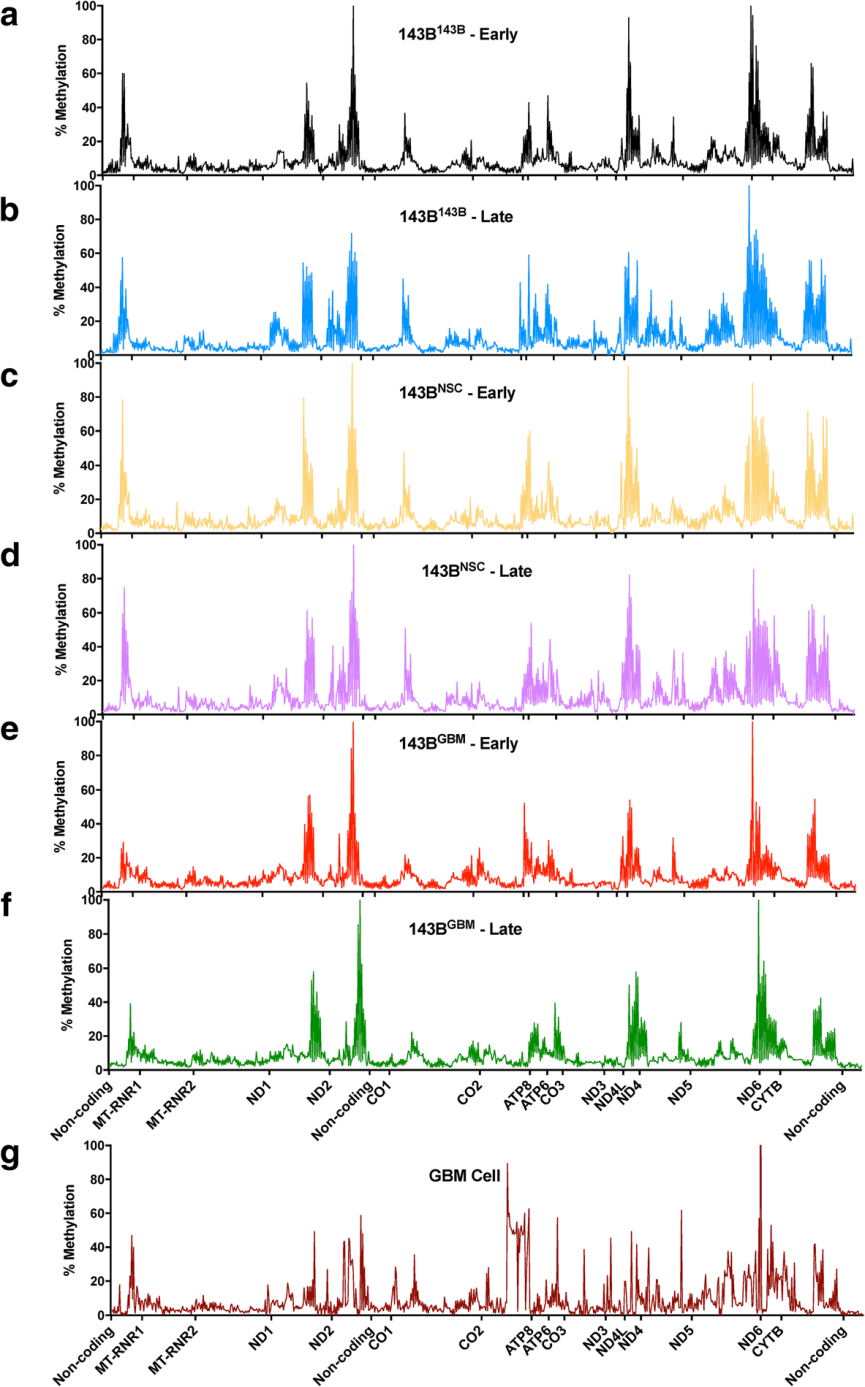
mtDNA methylation profiles determined by whole-mitochondrial genome bisulfite-sequencing. Whole-mitochondrial genome bisulfite-sequencing was applied to the purified mtDNA samples from **a** 143B^143B^ early tumors, **b** 143B^143B^ late tumors, **c** 143B^NSC^ early tumors, **d** 143B^NSC^ late tumors, **e** 143B^GBM^ early tumors, **f** 143B^GBM^ late tumors, and **g** GBM cells. Levels of methylation (% Methylation) at CpG sites across the whole genome are shown

We then determined the overall mtDNA methylation profiles for the 143B^143B^, 143B^GBM^, and 143B^NSC^ tumors and the GBM cells (Fig. 1). On average, the normalized methylation levels at CpG sites over the genome were 10% (Fig. 1), which is consistent with our previous finding using a MeDIP-qPCR assay and findings reported by others [17, 29].

### The changes in mtDNA methylation resulting from different mtDNA genotypes

Assessment of the 143B^143B^, 143B^GBM^, and 143B^NSC^ tumors at early and late stages showed that several CpG sites exhibited significant differential levels of DNA methylation (*p* < 0.05 and difference in DNA methylation > 5%) as a result of the different mtDNA genotypes being under the same nuclear genome (Table 1). Among the early tumors, there was only one site, which is located within the *ND5* region (13209), that showed lower levels of DNA methylation (7%) in the 143B^GBM^ early tumors when compared with the 143B^143B^ early tumors. mtDNA derived from cancer cells, namely 143B^143B^ and 143B^GBM^, showed more variable patterns compared with the 143B^NSC^ early tumors. 143B^NSC^ early tumors had higher levels of methylation than the 143B^143B^ early tumors at two CpG sites within the *CYTB* region: 15.75% higher for site 14578 and 12.04% higher for site 14802. Likewise, 143B^NSC^ early tumors showed higher levels of DNA methylation than the 143B^GBM^ early tumors at six sites, namely the same two sites identified in the 143B^143B^ early tumors: 12.46% for site 14578, 19.64% for site 14802; and at site 498 within the major non-coding region, site 9143 within the *ATP6* region, site 13209 within the *ND5* region, and site 14829 within the *CYTB* region. These sites were uniquely hyper-methylated in the 143B^NSC^ early tumors when compared with the 143B^GBM^ early tumors.

**Table 1:** Changes in mtDNA methylation resulting from different mtDNA genotypes

	Location of C	Strand	Annotation	143B^GBM^ vs. 143B^143B^		143B^NSC^ vs. 143B143B		143B^NSC^ vs. 143B^GBM^	
Early tumors	498	+	Non-coding					*	19.83%
	9143	+	ATP6					*	5.85%
	13,209	–	ND5	**	− 7.00%			***	7.54%
	14,758	+	CYTB			**	15.75%	**	12.46%
	14,802	+	CYTB			**	12.04%	**	19.64%
	14,829	+	CYTB					**	7.20%
Late tumors	3695	+	ND1	**	− 1.69%	**	10.58%		
	3705	+	ND1	***	− 7.57%			**	5.11%
	13,712	+	ND5					*	7.31%

*, **, *** indicate *p* values of < 0.05, 0.01, 0.001, respectively

Among the late tumors, similar patterns were observed, but fewer sites showed significant differences as a result of the different mtDNA genotypes. mtDNA derived from GBM cells had lower levels of DNA methylation than mtDNA derived for 143B cells at sites 3695 and 3705 within the *ND1* region. mtDNA derived from hNSC had higher levels of DNA methylation at site 3695 (10.58%) than 143B^143B^ late tumors, and at sites 3705 (5.11%) and 13712 (7.31%) than the 143B^GBM^ late tumors. Therefore, in the presence of the 143B nuclear genome, mtDNA derived from a tumor background tended to have lower levels of mtDNA methylation than mtDNA derived from a non-tumorigenic cell, such as hNSC, suggesting that mtDNA methylation is initially necessary to enforce the tumor phenotype.

### The changes in mtDNA methylation during tumorigenesis and assessment of regulatory regions using pyrosequencing

We also determined whether the patterns of mtDNA methylation changed during the process of tumorigenesis. Indeed, two sites were significantly modified and showed a decrease in levels of mtDNA methylation from the early stage to the late stage, namely site 3950 within the *ND1* region in the 143B^143B^ tumors and site 10399 within the *ND3* region in the 143B^NSC^ tumors (Table 2).

**Table 2:** Changes in mtDNA methylation resulting from tumorigenic progression

	Location of C	Strand	Annotation	*P* value	Difference
143B^143B^ Late vs. Early	3950	+	ND1	*	− 3.97%
143B^NSC^ Late vs. Early	10399	+	ND3	**	− 6.67%

*, ** indicate *p* values of < 0.05, 0.01, respectively

**Fig. 2:**
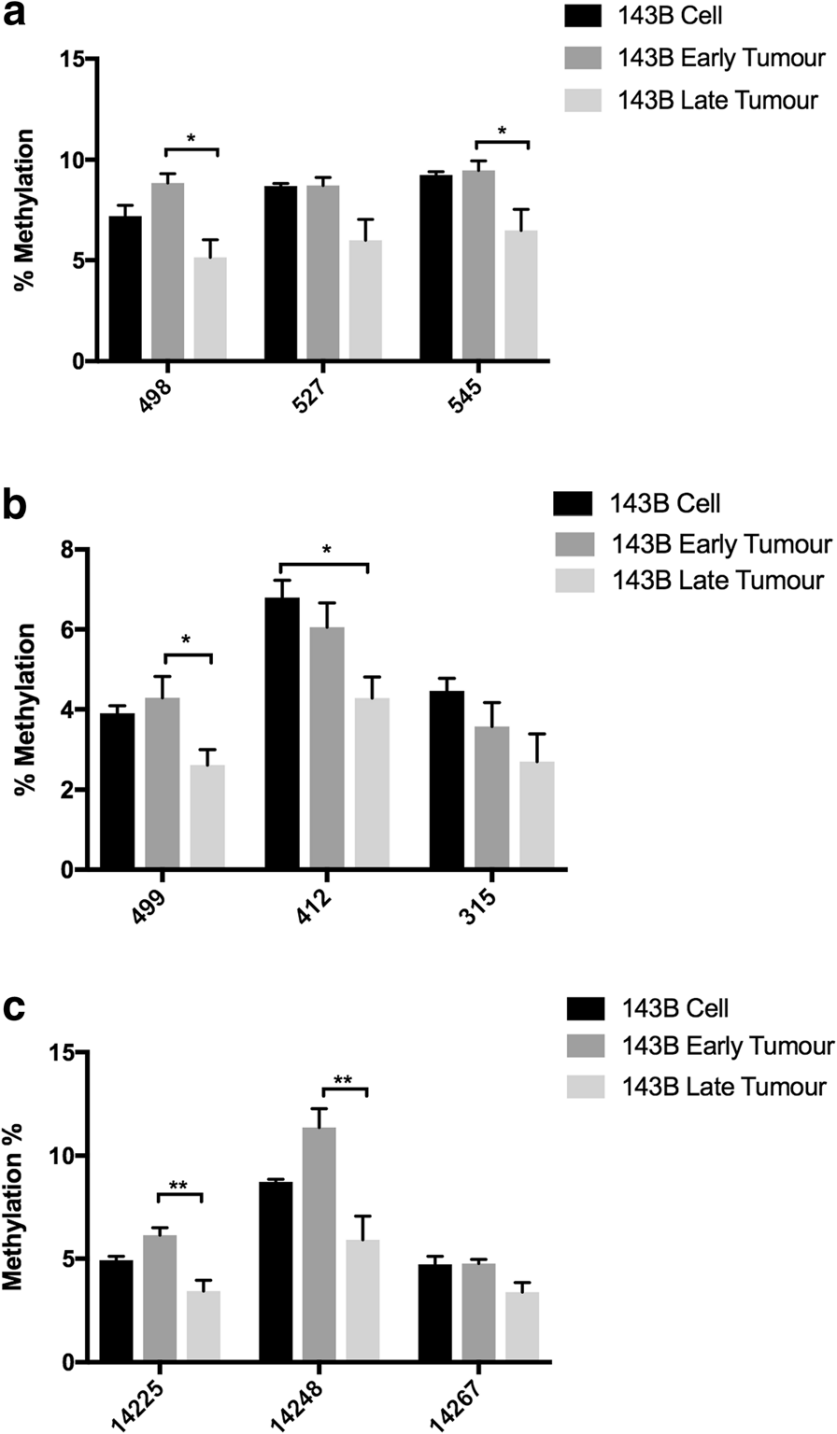
mtDNA methylation identified by pyro-sequencing. Levels of DNA methylation at CpG sites in **a** HSP, **b** LSP, and **c** *ND6* regions of 143B cells, 143B^143B^ early and 143B^143B^ late tumors were determined by pyro-sequencing. Statistical significance was determined by One-way ANOVA. Bars represent the mean of the percentage of DNA methylation (mean ± SEM; *n* = 3). * and ** indicate *p* values of < 0.05 and 0.01, respectively

As mtDNA copy number was significantly increased in all three tumor types during the process of tumorigenesis [5], the patterns across the D-loop, the site of interaction for the nuclear-encoded mtDNA replication factors, were further assessed using pyrosequencing on the 143B^143B^ cells, and 143B^143B^ early and 143B^143B^ late tumors. Pyrosequencing is a sensitive and accurate technique for determining DNA methylation levels, especially for the regions such as the D-loop that showed relatively lower levels of methylation (as seen in Fig. 1). Regions targeting HSP (Fig. 2a), LSP (Fig. 2b), and *ND6* (Fig. 2c; the only gene on the light strand) were successfully assessed. A decrease in levels of mtDNA methylation were observed at sites 498 and 545 for the HSP region, site 499 for the LSP region, and sites 14225 and 14248 for the *ND6* region from the early to the late stages. Furthermore, site 421 within the LSP region had significantly lower levels of DNA methylation in the late tumors compared with the cells.

### The impact of different nuclear genomes on mtDNA methylation

To understand the bi-directional control of the mitochondrial and nuclear genomes, it is necessary to investigate the impact of different nuclear genomes on mtDNA methylation. Thus, the mtDNA methylation profile of GBM cells was compared with 143B^GBM^ tumors as they possess the same mitochondrial genome (GBM), but are combined with different nuclear genomes (GBM and 143B).

GBM mtDNA methylation appeared to be extensively modulated under the different nuclear genomes (Table 3). In total, 25 sites were significantly different in the 143B^GBM^ early tumors compared with GBM cells, whereas 19 sites were significantly different in the 143B^GBM^ late tumors compared with GBM cells. Among these sites, 16 of them were commonly identified with similar differences in DNA methylation, including both higher and lower levels of DNA methylation in the tumors. Two sites within each of the *RNR2*, *ND1*, *ND2*, and *CO1* regions, and one site in each of the *ATP6/8*, *TR*, and *ND4L* regions showed higher levels of DNA methylation when GBM mtDNA is in combination with the 143B nuclear genome. Fewer sites showed significantly lower levels of DNA methylation in the 143B^GBM^ tumors, including one site in each of *CO1*, *ND4*, *ND5*, *ND6*, and *CYTB*. There were also uniquely differentially methylated sites identified in the 143B^GBM^ early and late tumors when compared with GBM cells. For the 143B^GBM^ early tumors, seven sites including one site within the D-loop region, three sites within *RNR2*, and one site in each of *ND1*, *CO1*, and *ND5* were uniquely changed. For the 143B^GBM^ late tumors, three sites including one site within *RNR1*, and one site in each of *ATP6* and *TG* were uniquely changed. These findings indicate that the patterns of mtDNA methylation vary depending on the nuclear genome and its cytoplasmic microenvironments.

**Table 3:** Changes in mtDNA methylation of the GBM genotype resulting from different nuclear genomes

Location of C	Strand	Annotation	143B^GBM^ Early vs. GBM cell		143B^GBM^ Late vs. GBM cell	
			*P*	Difference	*P*	Difference
170	+	Non-coding	*	7.49%		
1597	+	RNR1			*	4.96%
2719	−	RNR2	*	34.26%	**	27.12%
2810	−	RNR2	*	− 30.90%		
3022	−	RNR2	*	− 7.12%		
3061	−	RNR2	*	8.50%		
3094	−	RNR2	**	5.58%	**	4.74%
3945	−	ND1	*	6.56%		
3950	+	ND1	*	6.81%	*	9.48%
3965	+	ND1	*	4.92%	*	3.03%
4664	−	ND2	*	6.06%	*	4.63%
5110	+	ND2	*	5.61%	*	6.75%
6241	+	CO1	*	9.05%		
6850	+	CO1	*	− 12.72%	*	− 8.53%
7218	−	CO1	*	5.96%	*	4.56%
7336	−	CO1	*	5.02%	*	3.97%
8544	−	ATP8; ATP6	**	8.51%	*	7.57%
9008	+	ATP6			*	6.14%
10012	+	TG			*	8.09%
10169	−	ND3	*	12.33%		
10174	+	ND3	*	− 12.72%		
10426	−	TR	**	7.18%	*	8.24%
10584	+	ND4L	**	4.66%	*	5.54%
11765	−	ND4	*	− 5.74%	**	− 6.44%
13120	–	ND5	***	− 8.22%	***	− 7.38%
13938	+	ND5	*	− 34.96%		
14248	−	ND6	*	− 11.83%	*	− 12.43%
15043	+	CYTB	**	− 31.92%	*	− 31.28%

*, **, *** indicate *p* values of < 0.05, 0.01, 0.001, respectively

### The conversion of 5mC to 5hmC in mtDNA identified by MeDIP

**Fig. 3:**
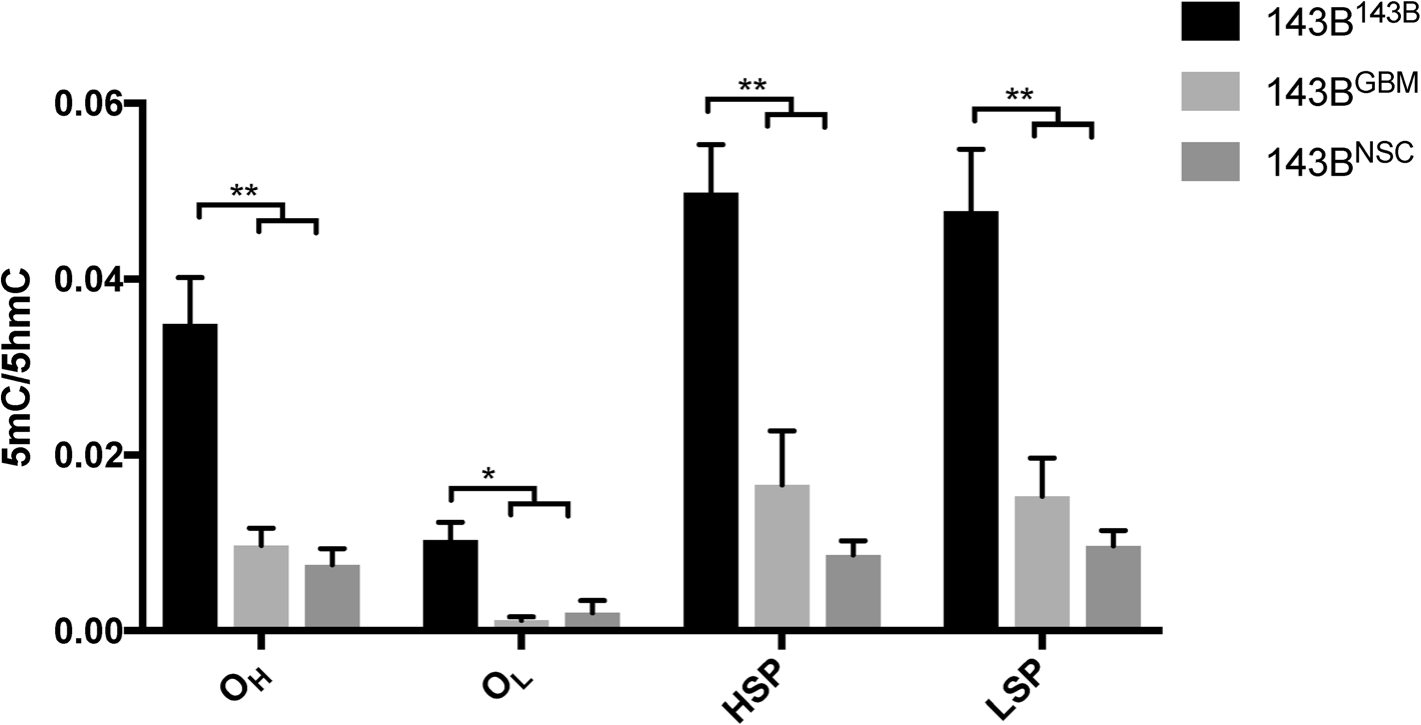
Levels of DNA methylation at different regulatory regions of the mitochondrial genome in late-stage 143B^143B^, 143B^GBM^, and 143B^NSC^ tumors. DNA methylation levels (5mC/5hmC) within regions of the mitochondrial genome were determined on purified mtDNA samples using MeDIP-qPCR. Statistical significance was determined between the 143B^143B^, 143B^GBM^, and 143B^NSC^ tumors by one-way ANOVA. Bars represent the mean of the relative quantification levels (mean ± SEM; n = 3). *, ** indicate *p* values of < 0.05, 0.01, respectively

Following the findings identified using whole-mtDNA bisulfite-sequencing, we applied MeDIP using antibodies to distinguish between 5mC, the methylated state, and 5hmC, the demethylated state, for 143B^143B^, 143B^GBM^, and 143B^NSC^ late-stage tumors. We focused on the key regulatory regions in mtDNA, namely O_H_, O_L_, LSP, and HSP, to further determine the levels of plasticity of mtDNA methylation. Generally, the levels of mtDNA methylation (5mC/5hmC) at O_H_, HSP, and LSP in the D-loop were highest in the 143B^143B^ tumors, followed by the 143B^GBM^ and 143B^NSC^ tumors (Fig. 3). O_L_ had lower levels of DNA methylation than the sites in the major non-coding region. Therefore, for each mtDNA genotype, DNA methylation presented in similar patterns across the genome with the major non-coding region having higher levels of methylation than the minor non-coding region.

**Fig. 4:**
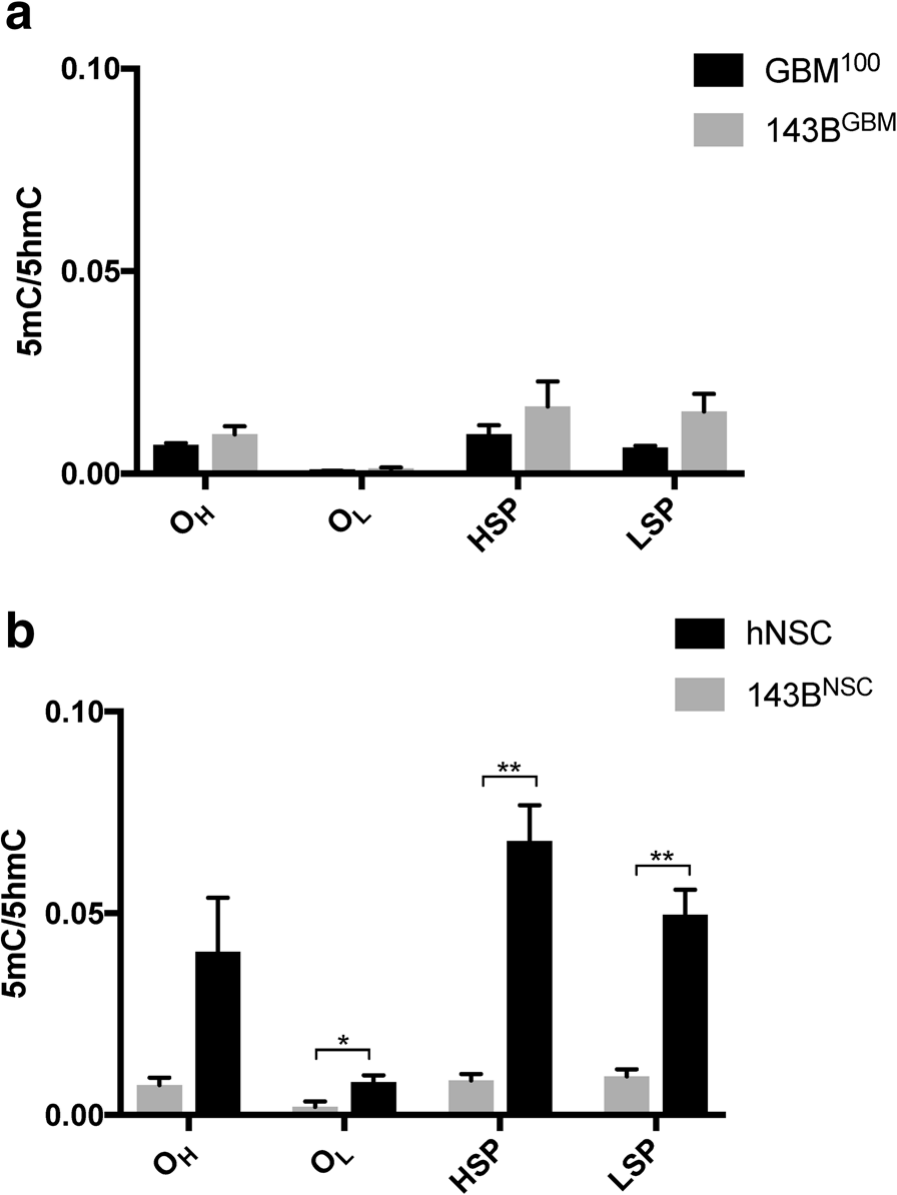
Levels of DNA methylation of the same mtDNA genotype under a different nuclear genome. **a** Levels of DNA methylation for GBM mtDNA in association with the GBM (GBM^100^) and 143B (143B^GBM^) nuclear genomes. **b** Levels of DNA methylation of hNSC mtDNA under the hNSC and 143B (143B^NSC^) nuclear genomes. DNA methylation levels (5mC/5hmC) were determined on purified mtDNA samples using MeDIP. Statistical significance was determined between each type by multiple *t* tests. Bars represent the mean of the relative quantification levels (mean ± SEM; *n* = 3). *, ** indicate *p* values of < 0.05, 0.01, respectively

We further investigated whether the conversion from 5mC to 5hmC of mtDNA derived from GBM tumors and hNSCs varied when under different nuclear genomes. Among the O_H_, O_L_, HSP, and LSP sites (Fig. 4), mtDNA from GBM tumors presented similar patterns of DNA methylation when they were under the GBM nuclear genome and the 143B nuclear genome. Interestingly, mtDNA from hNSCs had much higher levels of DNA methylation when they are under the tumorigenic 143B nuclear genome than the healthy hNSC nuclear genome. Therefore, mtDNA derived from the GBM tumor model could maintain its specific mtDNA methylation profiles under different tumorigenic nuclear genomes (143B or GBM), but mtDNA methylation derived from healthy cells tended to be reset by the tumorigenic nuclear genome.

### Different mtDNA methylation patterns resulted from the restoration of mtDNA copy number initiated from cells with varying levels of mtDNA

**Fig. 5:**
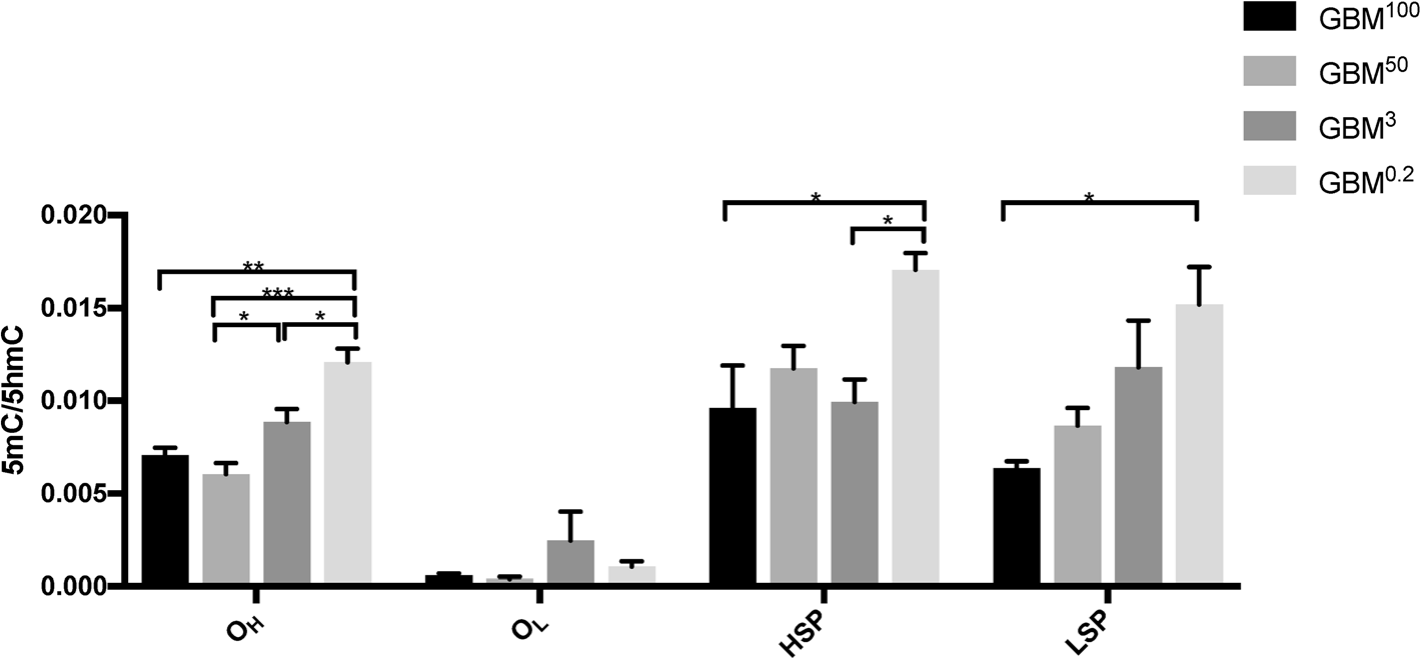
Levels of DNA methylation at different regions of the mitochondrial genome in GBM^100^, GBM^50^, GBM^3^, and GBM^0.2^ tumors. DNA methylation levels (5mC/5hmC) within regions of the mitochondrial genome were determined on purified mtDNA samples using MeDIP. Statistical significance was determined between the GBM^100^, GBM^50^, GBM^3^, and GBM^0.2^ tumors by one-way ANOVA. Bars represent the mean of the relative quantification levels (mean ± SEM; *n* = 3). *, **, *** indicate *p* values of < 0.05, 0.01, 0.001, respectively

Using GBM tumors formed from cells with varying levels of mtDNA, namely GBM^100^ (100% of original content), GBM^50^ (50% of original content), GBM^3^ (3% of original content), and GBM^0.2^ (0.2% of original content) tumors, we investigated the changes to mtDNA methylation (5mC/5hmC) after the restoration of mtDNA during tumorigenesis [6]. The GBM^3^ and GBM^0.2^ tumors exhibited significantly late onset and lower rates of tumor formation, as mtDNA copy number had to be restored to sufficient levels for the establishment of tumorigenesis [5, 6]. Once mtDNA had been sufficiently replicated to recover tumorigenic capability, mtDNA replication was restricted as tumor cells maintained low mtDNA copy number to support tumorigenesis. Here, we focused on the sites in the non-coding regions, namely O_H_, O_L_, HSP, and LSP (Fig. 5). For the origins of replication, only the O_H_ site presented differential DNA methylation patterns, as there were no significant differences at O_L_. GBM^0.2^ tumors had significantly higher levels of methylation than the other three cohorts, which likely restricts further replication after mtDNA copy number had been restored. GBM^3^ had significantly higher levels of methylation than the GBM^50^ tumors, which might contribute to the relatively faster onset in the formation of the GBM^50^ tumors [6]. GBM^0.2^ tumors also exhibited higher levels of methylation at HSP and LSP than the GBM^100^ tumors.

### Changes to the levels of mtDNA transcription and the correlation with mtDNA methylation

**Fig. 6:**
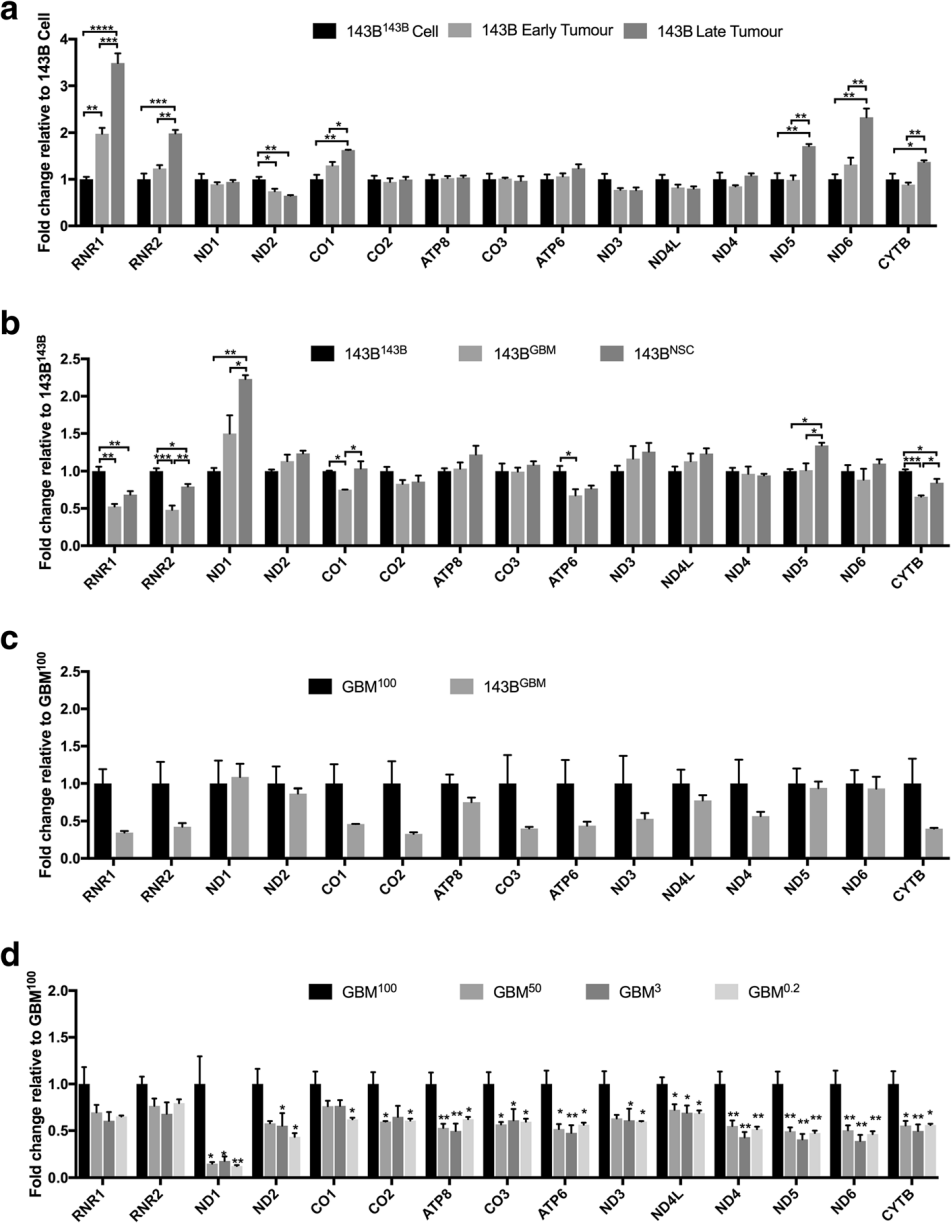
Significant differential expression of the mitochondrial genes using the Fluidigm array. Bars represent the mean of the relative quantification levels normalized to the control cohort (mean ± SEM; *n* = 3). Statistical significance was determined by one-way ANOVA for comparisons in **a** 143B^143B^ cells, and early and late-stage tumors; **b** 143B^143B^, 143B^GBM^, and 143B^NSC^ late-stage tumors; **d** GBM^100^, GBM^50^, GBM^3^, and GBM^0.2^ tumors; or by student *T* test for comparisons in **c** GBM^100^ and 143B^GBM^ tumors. *, **, ***, **** indicate *p* values of < 0.05, 0.01, 0.001, 0.0001, respectively

In addition to mtDNA replication, it is also of interest to assess the potential impact of mtDNA methylation on mtDNA transcription. Therefore, we performed a high-throughput qPCR Fluidigm array on the 143B cell lines and tumors and the HSR-GBM1 cell lines and tumors (Fig. 6). During the tumorigenesis of 143B^143B^ cells, there were increased levels of expression for *RNR1*, *RNR2*, *CO1*, *ND5*, *ND6*, and *CYTB*, whereas *ND2* showed a decrease (Fig. 6a). Among the 143B late-stage tumors harboring different mtDNA genotypes (Fig. 6b), genes within close proximity to the D-loop region, i.e., *RNR1*, *RNR2*, and *CYTB*, showed lower levels of gene expression in the 143B^GBM^ and 143B^NSC^ late-stage tumors than the 143B^143B^ late-stage tumors. However, *ND1* and *ND5* showed higher levels of gene expression in the 143B^NSC^ late-stage tumors than the other two groups. Moreover, overall, mtDNA derived from HSR-GBM1 cells showed lower levels of mtDNA expression under the 143B nuclear genome than under the HSR-GBM1 nuclear genome in late-stage tumors (Fig. 6c). To this extent, 2 rRNAs and 7 out of 13 ETC subunits (*CO1*, *CO2*, *CO3*, *ATP6*, *ND3*, *ND4*, and *CYTB*) were significantly downregulated in the late-stage 143B^GBM^ tumors than the GBM^100^ tumors. Interestingly, all of the ETC subunits were downregulated in the GBM tumors derived from cells depleted of mtDNA to varying levels (Fig. 6d).

**Fig. 7:**
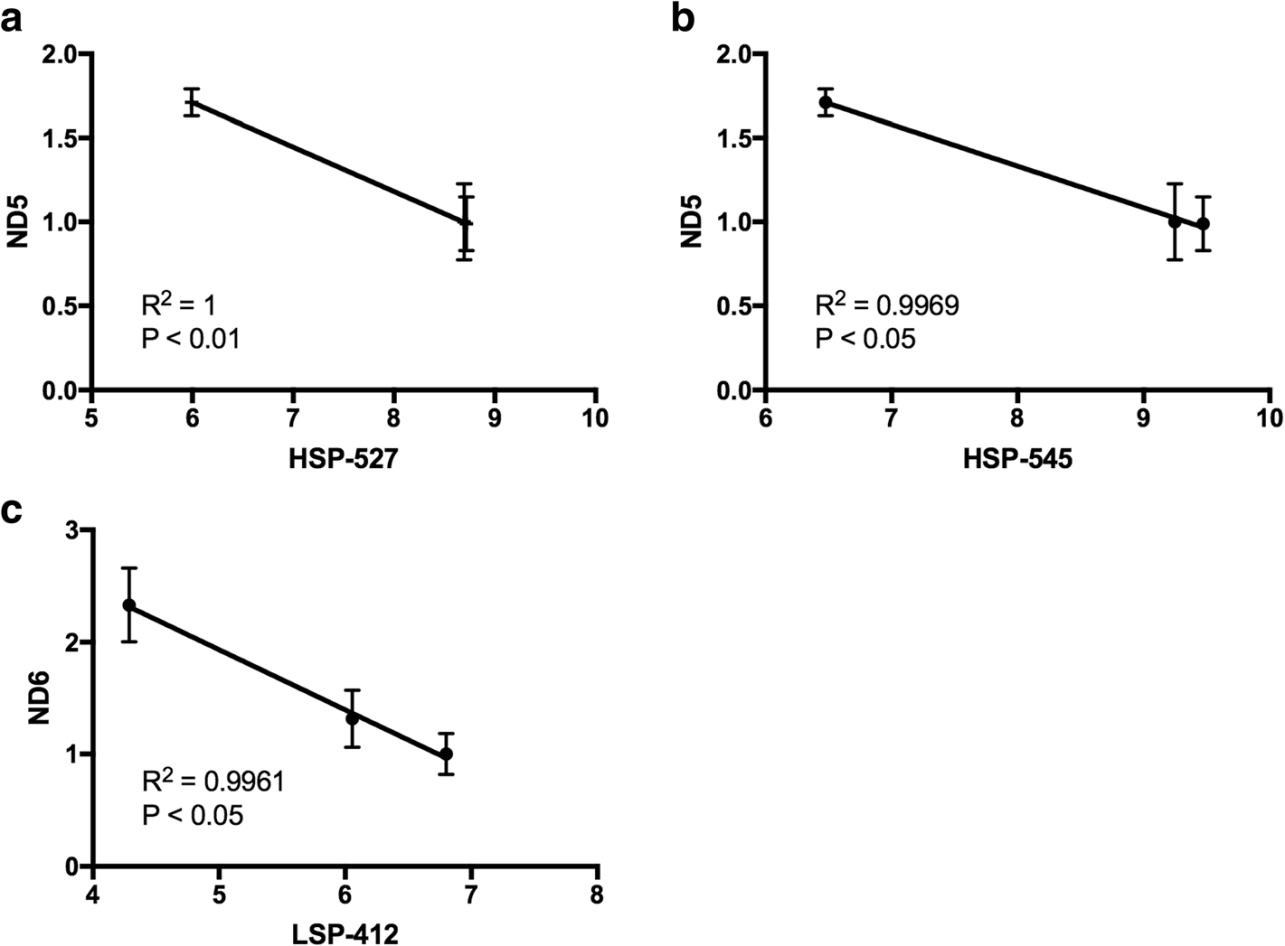
Pearson correlation tests between levels of mtDNA methylation and mtDNA transcription during 143B cell tumorigenesis. The level of *ND5* gene expression is correlated with the levels of DNA methylation at sites **a** 527 and **b** 545 of the HSP region. The level of *ND6* gene expression is correlated with the DNA methylation levels at **c** site 412 of the LSP region. Gene expression values (mean ± SEM; *n* = 3) were analyzed against the mean values of the methylation levels of each site. *R*^2^ and significance are shown in each plot

To determine whether there were correlations between the changes in mtDNA methylation and mtDNA expression, we performed correlation tests. During the progression of 143B cells to late-stage tumors, DNA methylation levels at sites 527 and 545 (HSP) significantly negatively correlated with the transcription of *ND5*, while the level of DNA methylation at site 412 (LSP) significantly negatively correlated with the transcription of *ND6* (Fig. 7). No significant correlations were identified at other sites and regions of the D-loop among the 143B tumors with different genotypes, between 143B^GBM^ and GBM^100^ tumors, and among GBM tumors formed from cells with varying levels of mtDNA content.

## Discussion

Our findings demonstrate that mtDNA methylation adds another layer to the epigenetic control of mtDNA copy number in tumor cells. It has been previously reported that the DNA methylation status at key regions of the nuclear-encoded mtDNA replication factors, namely exon 2 of *POLG* and exon 8 of *TOP1MT*, are related to mtDNA copy number [4, 7, 8, 16, 19]. Since the findings that mtDNA demethylation can also be induced by DNA demethylation agents that likely contributes to the upregulation of mtDNA copy number in tumor cells [16], it is important to determine the potential role that mtDNA methylation plays in regulating mtDNA replication. However, the presence of mtDNA methylation has been hotly debated over several decades with its role in mitochondrial genetics remaining largely undetermined [13, 17, 30].

In this study, we employed a technical pipeline to remove ambiguity associated with the results. Firstly, we used purified populations of mtDNA to avoid the noise arising from the mtDNA pseudogenes present in the nucleus [16]. Secondly, it is the first time that genotype-specific analysis has been applied to assess mtDNA methylation, which is a necessary step given that mtDNA is susceptible to variants in, for example, cancers [20] and different mtDNA haplotypes are differentially predisposed to various forms of cancer and other diseases [20, 31–33]. Indeed, we identified two CpG sites at nt 9053–9054 and nt 11911–11912 that were gained by the 143B and GBM mtDNA genotypes compared with the hNSC genotype. Thirdly, the strategy of using linearized positive and negative controls for each genotype generated by long PCR and treated in the presence and absence of a DNA methylating enzyme, respectively, provided a baseline for data normalization. This enabled the full potential of each CpG site among the population of mtDNA in the cells and tumors to be determined. Lastly, whole-genome bisulfite sequencing achieved significantly high coverage (> 500-fold coverage on average) to avoid possible overestimation of mtDNA methylation levels resulting from low coverage of sequencing reads (< 250-fold) as argued in [13]. In addition to the issue of sequencing coverage, the circular and supercoiling structure of mtDNA has been considered as another concern in inhibiting bisulfite treatment and causing overestimation in mtDNA methylation [13]. To this extent, the purified mtDNA samples and the linearized long PCR control samples were firstly fragmented before bisulfite conversion to overcome the potential effects of supercoiling, an approach we previously employed when assessing mtDNA methylation by MeDIP-Seq [16]. As a result, our findings were consistent with published results [17, 29]. Moreover, the use of 5mC- and 5hmC-specific antibodies in MeDIP enabled further analysis of the extent of mtDNA methylation by determining the degree of active demethylation in the samples, namely the transition from 5mC to 5hmC, which is not distinguishable following bisulfite conversion. Nevertheless, pyrosequencing, a highly sensitive method for determining the levels of DNA methylation at specific sites, further identified the subtle changes in the key regulatory regions of the early and late tumors.

In our studies, we have used a model of osteosarcoma, namely 143B cells, and early- and late-stage tumors derived from these cells that possessed the same chromosomal backgrounds but different mtDNA genotypes, i.e., 143B^143B^, 143B^GBM^, and 143B^NSC^ cells and tumors. This enabled us to investigate the patterns of mtDNA methylation between different genotypes at different stages of tumor progression. Indeed, these tumors have been shown to have different patterns of mtDNA replication during tumorigenesis [5]. Even though an increase in mtDNA copy number was observed during tumor progression across all three types of tumors, the 143B^NSC^ and 143B^GBM^ tumors gained significantly greater mtDNA copy number at the later stage compared with the 143B^143B^ tumors [5]. Therefore, 143B tumors served as excellent models to investigate whether mtDNA methylation regulates mtDNA copy number.

We firstly identified genotype-specific mtDNA methylation profiles. In the presence of the 143B nuclear genome, mtDNA derived from cancer cells, i.e., 143B and GBM mtDNA, tended to have lower levels of mtDNA methylation than hNSC mtDNA. All of the differentially methylated CpG sites within the non-coding region, *ATP6*, *CYTB*, *ND1*, and *ND5* identified in the early and late tumors had higher levels of DNA methylation in the 143B^NSC^ tumors than the 143B^GBM^ and 143B^143B^ tumors. Moreover, there were three sites that were significantly more hypo-methylated in the 143B^GBM^ tumors than the 143B^143B^ tumors, which indicates that different mtDNA genotypes from different cancer backgrounds could also lead to different patterns of mtDNA methylation and indicates that the mitochondrial genome is somehow responsible for establishing its methylation status independent of the nucleus. Indeed, different cancers have been associated with different combinations of mtDNA mutations [33, 34], which could contribute to their tumor-specific profiles and further emphasize the importance of the genotype-specific context of mtDNA in investigating mtDNA methylation. The findings identified by MeDIP further indicate that, among these methylated CpG sites, there were different rates of 5mC to 5hmC conversion. Even though 143B^NSC^ tumors had higher levels of mtDNA methylation at several CpG sites, they exhibited greater levels of demethylation, especially within the D-loop region. 143B^143B^ tumors maintained the highest levels of 5mC over 5hmC among the three types of tumors. This could be further associated with the changes in mtDNA copy number and that the higher levels of 5mC could contribute to the relatively restricted replication of mtDNA in the 143B^143B^ tumors. Moreover, changes to mtDNA transcription were also observed between different mtDNA genotypes. However, it appears that not just mtDNA methylation is affected by mtDNA genotype. In the 143B model, mtDNA genotype influenced the expression of chromosomal genes, which also gave clear indications of the genes specific to osteogenic tumorigenic initiation and maintenance [5].

During the process of tumorigenesis, decreases in the levels of mtDNA methylation were observed at two gene regions, namely site 3950 within the *ND1* region in 143B^143B^ tumors and site 10399 within the *ND3* region in the 143B^NSC^ tumors. *ND1* and *ND3* encode subunits of complex I of the ETC and are associated with mutations in cancers [33, 34], and changes in mtDNA replication mediated by surrounding mtDNA methylation sites could potentially affect the frequency of these mutated copies. Indeed, there were significant increases in mtDNA copy number among these tumors from the early to the late stages [5]. Likewise, a decreasing trend in DNA methylation was observed among the CpG sites within the *ND6*, and the HSP and LSP regions from the early stage (Fig. 2), which could potentially contribute to the active binding of the mtDNA replication factors to mtDNA, as is the case for *POLG* where it has been shown that the methylation status of exon 2 is associated with the binding of RNA polymerase II [19]. As mtDNA transcripts are primarily polycistronic and subsequently processed by tRNA punctuation [35, 36], we observed gene-specific changes to the mitochondrial genes but not general trends resulting from the regulation of mtDNA methylation. To this extent, changes to gene expression levels of *ND5* and *ND6* were significantly correlated with methylation levels of HSP and LSP, respectively. This suggests that there is likely to be an impact of DNA demethylation during 143B^143B^ tumorigenesis which reflects on the upregulation of *ND5* and *ND6* expression in late-stage tumors. A similar mechanism may exist during development as mtDNA copy number changes in synchrony with changes to nuclear gene expression at key stages of cellular differentiation [2, 19, 28].

In order to investigate whether the levels of mtDNA methylation change under different nuclear genomes, we also assessed GBM cells through the same experimental pipeline. GBM cells had a distinct mtDNA methylation landscape from the various combinations of 143B tumors. Indeed, GBM mtDNA had 25 CpG sites and 19 CpG sites were differentially methylated when compared with the methylation profiles of 143B^GBM^ early and late tumors, respectively. Sixteen of these CpGs were differentially methylated in the 143^GBM^ tumors. These sites are located across the whole genome, including *RNR2*, *ND1*, *ND2*, *CO1*, *ATP6*/*8*, *TR*, *ND4L*, *ND4*, *ND5*, *ND6*, and *CYTB*. A number of findings that have focused on either specific regions or the whole mitochondrial methylome has identified regions with different patterns of DNA methylation, as shown in mouse and human tissues (brain, liver, breast, and embryonic stem cells) and associated with disease (aging, colon cancer, 143B, and GBM) [16–18, 37–40]. These findings not only suggest that these regions can be differentially methylated in different scenarios but also indicate a strong relationship between the mitochondrial methylome and tissue-specific mtDNA copy number in synchrony with the tissue-specific nuclear genome background. Both hyper- and hypo-methylation were observed, but over 60% of all the identified sites were hyper-methylated when the GBM mtDNA was in the presence of the 143B nuclear genome. Moreover, the 5mC/5hmC rates remained at similar levels across the key regulatory sites in the non-coding regions. These changes to mtDNA methylation were not correlated with the downregulation of mtDNA transcription observed in the 143B^GBM^ late tumors. Interestingly, hNSC mtDNA had a greater rate of conversion for 5mC to 5hmC in the D-loop region (O_H_, LSP, and HSP) when in the presence of the 143B nuclear genome, as indicated by lower 5mC/5hmC values. This indicates that mtDNA methylation derived from healthy cells tended to be reset by the tumorigenic nuclear genome and the availability of cytoplasmic metabolites. Indeed, the communication between the two genomes is highlighted by the mtDNA replication and transcription factors being nuclear-encoded factors that translocate to the mitochondrion. To this extent, when, for example, HSR-GBM1 cells undergo global DNA demethylation induced by 5-Aza and VitC, not only are there global changes to the expression of nuclear-encoded genes associated with tumorigenesis but they exhibit improved mtDNA replication by modulating the levels of DNA methylation and upregulating the expression of the mtDNA replication factors [16]. In the cytoplasmic environment, the TET enzymes, which modulate the conversion from 5mC to 5hmC, are regulated by the isocitrate dehydrogenase (IDH) in the citric acid cycle (TCA), which takes place in the mitochondrion [41, 42] through the generation of α-ketoglutarate, a co-factor that facilitates TET activity. However, it has been reported that downregulation of the hypoxia regulator HIF1α promotes IDH in osteosarcoma [43], which provides an explanation for the greater conversion from 5mC to 5hmC mediated by TET enzymes. Moreover, s-adenosylmethionine is produced as a result of one-carbon metabolism and acts as the universal methyl group donor for DNA methylation with abnormal levels reported in cancer cells [44] and could also contribute to the variable levels of mtDNA methylation that take place during tumorigenesis. Collectively, these findings indicate that the nuclear genome and the cytoplasmic microenvironment exert a strong influence on the mitochondrial genome and its methylated status.

Another interesting perspective in our understanding of how mtDNA methylation regulates copy number is the adaptive nature of mtDNA methylation during the formation of tumors from mtDNA-depleted cells. mtDNA copy number was restored to similar levels in late-stage GBM tumors [6], which explains why the GBM^3^ and GBM^0.2^ cells took longer to initiate tumor formation [5, 6]. Moreover, various mtDNA variants have been identified within GBM and the accumulation of these variants is proportional to the amount of mtDNA being restored [33], which makes these tumors interesting models to understand GBM-specific replication of mtDNA. GBM^0.2^ tumors had significantly higher levels of DNA methylation within the D-loop region than the other three cohorts, which likely further restricts replication of mtDNA after they had re-established their original copy number. Indeed, once mtDNA had been sufficiently replicated to recover tumorigenic capability, mtDNA replication was restricted as tumor cells maintained low mtDNA copy number to support tumorigenesis. Consequently, instead of primarily using OXPHOS, tumors cells mainly rely on aerobic glycolysis for energy production, which switches the metabolic profiles to prioritize cellular proliferation and prevents differentiation from taking place [4] Likewise, GBM^3^ tumors had significantly higher levels of methylation than the GBM^50^ tumors, which likely contributes to the relatively faster onset in the formation of the GBM^50^ tumors [6]. Furthermore, mtDNA transcription was generally downregulated in the GBM tumors formed from the cells with varying levels of mtDNA depletion, which potentially reflected the impact of mtDNA methylation on mtDNA transcription.

## Conclusions

In all, by applying robust experimental pipelines to assess the levels of DNA methylation in 143B and GBM cells and tumor models, we not only confirmed the presence of mtDNA methylation but also determined the levels of mtDNA methylation in different scenarios. Firstly, different mtDNA genotypes under the same nuclear genomic background modulated levels of mtDNA at several sites across the mitochondrial genome, whereby higher levels of 5mC are related to the relatively restricted expansion of mtDNA copy number in the 143B^143B^ tumors. Secondly, the same mtDNA genotype behaved differently under the influence of different nuclear genomes to regulate mtDNA methylation. Thirdly, levels of mtDNA methylation tended to decrease during tumor progression, which could potentially contribute to the increases in mtDNA copy number observed in these tumors. Changes to the levels of DNA methylation correlated with transcriptional changes to *ND5* and *ND6* during the tumorigenesis of 143B^143B^ cells, which potentially indicates the impact of mtDNA methylation on primary polycistronic transcription, and that mtDNA methylation is a dynamic process that could take place during development. Lastly, after tumors had restored sufficient mtDNA to initiate tumorigenesis, higher levels of 5mC over the D-loop were acquired to potentially restrict further replication of mtDNA. Collectively, mtDNA methylation adds another layer to the epigenetic control of mtDNA replication.

## Methods

### Cell culture

HSR-GBM1 cells were cultured in complete neural stem cell media consisting of Dulbecco’s modified Eagle medium/Nutrient Mixture Media (DMEM/F-12; Thermo Fisher Scientific, MA, USA), 2% StemPro neural supplement (Thermo Fisher Scientific), basic fibroblast growth factor (bFGF; 20 ng/mL; Merck Millipore, MO, USA), and epidermal growth factor (EGF; 20 ng/mL; Merck Millipore) at 37 °C in 5% CO_2_ and 95% humidity. The mtDNA content of HSR-GBM1 cells was depleted to varying levels, namely 50%, 3%, and 0.2% of the original mtDNA content, by additionally culturing with 2′-3′-dideoxycytidine (ddC; 10 μm Sigma-Aldrich, MO, USA) and uridine (50 mg/mL; Sigma-Aldrich) [6].

143B cell lines possessing different mtDNA genotypes derived from HSR-GBM1 cells and hNSCs were previously generated, namely 143B^GBM^ and 143B^NSC^, as described in [5]. Briefly, 143B cells were completely depleted of mtDNA by long-term exposure to ethidium bromide and then repopulated with donor mtDNA through fusion with enucleated hNSC and HSR-GBM1 cytoplasts, as previously described [45, 46]. To prepare the donor cytoplasts, 3 × 10^6^ hNSC or HSR-GBM1 cells were resuspended in 10 mL of SD-DMEM, 10 mL of Percoll solution (Sigma), 200 μL penicillin/streptomycin, and 2 mg/mL cytochalasin B (Sigma). Cells were then enucleated by centrifugation at 20000 rpm using a SS-34 fixed angle rotor (Thermo Scientific, MA, USA) for 70 min at 27 °C. Then, 7.5 mL of the Percoll/media interface was transferred to a 15 mL tube and 7.5 mL of fresh SD-DMEM was added to the cell suspension and centrifuged at 4400 rpm for 5 min. The supernatant was then removed and the cell pellets were resuspended in 10 mL of SD-DMEM. Further, 1 × 10^6^ mtDNA depleted 143B cells were mixed with 10 mL cell suspension of hNSCs or HSR-GBM1 cells and centrifuged at 10,000 rpm using the SS-34 fixed angle rotor at 27 °C for 10 min. Cell pellets were collected and overlain with 500 μL of cell culture grade polyethylene glycol (PEG; Sigma) for 1 min. PEG was immediately removed and the cell pellets were resuspended in 10 mL of SD-DMEM to complete fusion. The fusion mixtures were then cultured in SD-DMEM at 37 °C in 5% CO_2_ and 95% humidity. After 24 h, the fusion mixtures were transferred to the selection media, which consisted of RPMI medium (Life Technologies), 5% FBS, 2 mM GlutaMax, 1% penicillin/streptomycin, and 20 μg/mL 5-bromo-2-deoxyuridine (BrdUrd; Sigma). The medium was replaced every 2 days. After 7–14 days, propagated cell colonies were isolated and expanded in the selection media. Once established, the 143B^143B^, 143B^GBM^, and 143B^NSC^ cell lines were then cultured in standard DMEM (SD-DMEM) consisting of DMEM, 10% (*v*/*v*) FBS, sodium pyruvate (10 mM), GlutaMax (2 mM), and 1% (*v*/*v*) penicillin/streptomycin (Thermo Fisher Scientific) at 37 °C in 5% CO_2_ and 95% humidity [5].

### Xenograft models

GBM tumors were previously generated from HSR-GBM1 cells possessing 100%, 50%, 3%, and 0.2% of their original mtDNA content, namely GBM^100^ (non-depleted), GBM^50^, GBM^3^, and GBM^0.2^ tumors, respectively, as described in [6]. 143B tumors were previously generated from 143B^143B^, 143B^GBM^, and 143B^NSC^ cell lines, as described in [6]. Briefly, 0.5 million of the depleted and non-depleted HSR-GBM1 cells, or 1 million of each type of the three 143B cell lines in 100 μL of Matrigel (Corning, New York, USA) were inoculated subcutaneously into the right flank of 8-week-old, female BALB/c nude mice. Early-stage tumors were collected when the tumors were first palpable, i.e. at a volume of ~ 50 mm^3^, whereas the late-stage tumors were collected at the end point of tumor progression (~ 800 mm^3^). Tumor growth rates and volumes were reported in [5, 6].

### DNA and RNA extraction

Total genomic DNA and RNA were extracted from cultured cell pellets or tumor tissues using the DNeasy Blood & Tissue Kit and RNeasy Mini Kit (Qiagen, CA, USA), respectively, according to the manufacturer’s protocols with minor modifications. The DNA samples were treated with 3 μL of RNase A (Qiagen) at room temperature and Proteinase K (20 μg/μL; Bioline, London, UK) at 65 °C for 10 min. The RNA samples were treated with DNase I (3 kunitz units/μL; Qiagen) in the presence of RDD buffer at room temperature for 20 min.

### Purification of mtDNA from tumor tissues and cells

To eliminate mtDNA pseudogenes present in the nuclear genome, mtDNA was purified from tumor tissues and cells, as described in [16]. Cells (~ 10 million) and tumor tissues (~ 20 mg) were resuspended in 5 mL of solution A (20 mM HEPES-KOH, pH 7.6, 220 mM Mannitol, 70 mM sucrose, 1 mM EDTA) and 2 mg/mL BSA was added. The suspension was incubated on ice for 15 min to facilitate swelling and then homogenized at 4 °C using a 5 mL Potter-Elvehjem tissue grinder set (Wheaton, USA) for 50 repetitions. The homogenate was centrifuged at 800 g for 10 min to remove cell debris and nuclei. The supernatant was then centrifuged at 10,000 g for 20 min at 4 °C to pellet the mitochondrial fraction. To further remove nuclear DNA, the pellet was resuspended in 175 μL of solution B (solution A without EDTA) with DNase I (30 kunitz units; Qiagen) and incubated at 37 °C for 30 min. Then, 1 mL of solution A was then added to stop DNase activity and the suspension was centrifuged at 10,500 g for 20 min at 4 °C. The supernatant was discarded and the mitochondrial pellet was resuspended in 200 μL of lysis buffer (50 mM Tris-HCl, pH 8.0, 10 mM EDTA, and 1% SDS) with 1 μL of proteinase K (20 mg/mL, Bioline). The suspension was then incubated at 50 °C for 60 min. mtDNA was purified using the DNeasy Blood & Tissue Kit (Qiagen), according to the manufacturer’s protocol.

### Generation of negative and positive controls for mtDNA bisulfite sequencing by long PCR

To generate experimental controls for whole-mitochondrial-genome bisulfite sequencing, long PCR products from mtDNA were generated as negative controls. PCR products do not maintain methylation modifications and, therefore, are deemed to be unmethylated. Two overlapping mtDNA sequences spanning the whole mitochondrial genome were generated by long PCR and combined in equivalent concentrations as the negative control. Each long PCR reaction contained 1 unit of Platinum Taq High Fidelity (Thermo Fisher Scientific) in High Fidelity PCR buffer, 100 mM MgSO_4_, 1 mM dNTPs (Bioline), and 10 μM of each forward and reverse primer (Additional file 4) in a total volume of 50 μL. PCR cycling profiles were initiated at 94 °C for 2 min, followed by 35 cycles of 94 °C for 15 s, 63 °C for 30 s, and 68 °C for 8 min, 45 s. Long PCR products were then purified using a PCR QIAquick PCR Purification Kit (Qiagen), according to the manufacturer’s instructions. Importantly, in order to generate mtDNA-genotype-specific long PCR controls, DNA samples purified from 143B^143B^, 143B^GBM^, and 143B^NSC^ tumors were used as the respective templates.

Similarly, the positive controls comprised the two long PCR products that had been combined and underwent DNA methylation treatment using an in vitro CpG methyltransferase, *M.SssI* (New England Biolabs, MA, USA), according to the manufacturer’s instructions. Briefly, 1 μg of combined long PCR products was treated with 4 units of the *M.SssI* enzyme in the presence of 160 μM S-adenosylmethionine (SAM) at 37 °C for 4 h. The reaction was then terminated by incubating the products at 65 °C for 20 min. Long PCR products were then purified from the mixture using the PCR QIAquick PCR Purification Kit (Qiagen), according to the manufacturer’s instructions.

### Bisulfite sequencing

Purified mtDNA samples and control samples generated using long PCR were submitted to the MHTP Medical Genomics Facility (Clayton, Australia) to perform bisulfite sequencing. Libraries were prepared using the Zymo Pico Methyl-Seq™ Library Prep Kit following protocol 1.0.0 (Zymo Research, CA, USA), which is compatible with Illumina’s TruSeq chemistries. Briefly, all samples were processed with 50 ng of DNA based on measurements generated by Qubit Fluorometric Quantitation (Thermo Fisher Scientific). Samples underwent 16 cycles of amplification, as recommended by the manufacturer’s protocol. Samples then underwent random fragmentation to linearize mtDNA molecules to overcome the overestimation of DNA methylation caused by the circular and supercoiling structure of mtDNA molecules, as raised in [13]. Samples then underwent bisulfite conversion, random priming, and addition of sequencing indexed adaptors, according to the manufacturer’s protocol. Libraries were pooled in equivalent molar ratios. Furthermore, 14 pM of a single pool was assessed by qPCR and then used for generating the sequencing clusters. Size and concentration of the libraries were checked using a 2100 Bioanalyzer with the High Sensitive DNA Kit (Agilent). Then, 150-bp paired-end sequencing was performed on the Illumina MiSeq v2 platform.

### mtDNA-genotype-specific analysis for bisulfite sequencing

Firstly, adaptors and poor-quality reads were cleaned from raw sequences using the TrimGalore program (v0.4.5; http://www.bioinformatics.babraham.ac.uk/projects/trim_galore/) in the paired-end mode with 10 bp trimmed at both ends. Trimmed sequences were checked by the FastQC program to ensure the quality of the reads and for complete removal of any remaining adaptors.

Since mtDNA is known to be susceptible to variants in both non-coding and coding regions in cancer [20], it is important to select matching mitochondrial genotypes as the reference genomes for the mapping of mtDNA sequences [27]. We had previously sequenced the 143B^143B^, 143B^GBM^, and 143B^NSC^ mitochondrial genomes and these are available in GenBank under accession numbers KT946592, KT946593, and KT946594, respectively [5]. The sequences were mapped to their corresponding mitochondrial genomes using the Bismark Package (v0.19.0) with the paired-end mode set to the parameter of “-bowtie2-non_directional-bam” [47]. PCR duplicates were then removed using the function of “deduplicate_bismark” with the parameters “-p-bam”. The coverage of the methylated and unmethylated CpG and non-CpG sites in the mitochondrial genome were constructed using the function of “bismark_methylation_extractor” with the parameters “-p-comprehensive-merge_non_CpG-bedgraph-CX-counts-cytosine_report-CX”.

CpG sites that were covered by a minimum of ten reads in all of the negative and positive long PCR samples generated for each genotype were kept for further analysis. The differences in the percentage methylation between the negative and the positive long PCR samples were determined to be the full potential (100%) for each of the CpG sites in each mtDNA genotype [48]. These full potentials were then applied to normalize the levels of DNA methylation of the respective tumor samples at their corresponding CpG sites. Normalized levels of DNA methylation then underwent comparison using the one-way ANOVA method in Prism 7.0 software (GraphPad Software, CA, USA).

### Immunoprecipitation of methylated DNA

Purified mtDNA underwent methylated-DNA-immunoprecipitation (MeDIP), as described in [49]. Briefly, 5 μg of purified mtDNA was sheared into 200–1000 bp fragments by the Covaris Adaptive Focused Acoustics (AFA™) S220 system. The DNA was denatured by heating at 95 °C for 10 min and then cooled on ice for 5 min, which also avoided the potential issue raised by the circular, supercoiled structure of mtDNA [13]. Then, 1.5 μg of anti-5mC or anti-5hmC antibody (Active Motif, CA, USA) were added to 3 μg of DNA fragments in the presence of 20 μL of Dynabeads® Protein G (Thermo Fisher Scientific) in 500 μL of IP buffer (100 mM sodium phosphate, pH 7.0; 1.4 M NaCl;0.5% Triton X-100). The suspension was incubated at 4 °C for 16 h under rotation. The beads were collected on a magnetic particle concentrator (Thermo Fisher Scientific) and washed with 1 mL of IP buffer three times. The beads were then resuspended in 250 μL proteinase K digestion buffer (50 mM Tris-HCl, pH 8.0; 10 M EDTA, pH 8.0; 1.0% SDS) with 10 μL of proteinase K (20 mg/mL; Bioline) and incubated on a thermo-shaker at 50 °C for 3 h. The supernatant was then collected into a new tube. DNA was purified from the elutant using the QIAquick PCR Purification Kit (Qiagen) and collected in 50 μL of autoclaved Milli-Q H_2_O. Levels of DNA methylation for the regions of interest were quantified by qPCR on the Rotor-Gene 3000 machine under primer-specific conditions (Additional file 4). The ratio of 5mC/5hmC was determined.

### Pyrosequencing

DNA samples were submitted to the Australian Genome Research Facility (Perth, Australia) to perform pyrosequencing. DNA samples were firstly converted using the Epitect Bisulphite Kit (Qiagen), according to the manufacturer’s instructions. Bisulfite-converted primers were designed using the PyroMark Assay Design software for the regions of interest (Additional file 4). The regions of interest were amplified using the PyroMark PCR Kit (Qiagen) with biotin-labeled primers that were purified using HPLC (Additional file 4). PCR products were then immobilized to the Streptavidin Sepharose High Performance beads (GE Healthcare Life Sciences, MA, USA). PCR products with the beads underwent denaturation to anneal with the pyrosequencing primers (Additional file 4). Pyrosequencing was then performed on a PyroMark 24 PyroSequencing system (Qiagen), according to the manufacturer’s instructions. DNA methylation levels for each CpG site were exported using PyroMark Q24 software. Data that were identified as having failed bisulfite conversion and QC were ignored for further analysis. Statistical differences were determined amongst groups using One-way ANOVA (comparison between three groups) or Student *T* test (comparisons between two groups) (*n* = 3; mean ± SEM).

### mtDNA gene expression analysis using the Fluidigm platform

The expression of the 13 subunit genes of the ETC and 2 ribosomal RNAs encoded by the mitochondrial genome was determined using the Fluidigm platform, as described in [50]. Briefly, cDNA was synthesized from 1 μg of total RNA for each cell line or tumor sample using the Superscript III First-Strand synthesis system (Thermo Fisher Scientific), according to the manufacturer’s instructions. Taqman gene expression primers (Additional file 5) were pooled and diluted in C1 DNA suspension buffer to a final concentration for each primer of 180 nM. Each cDNA sample and a non-template control underwent pre-amplification for 14 cycles with the Taqman PreAmp Master mix (Thermo Fisher Scientific) and the pooled Taqman primers. Products were then diluted fivefold with C1 DNA suspension buffer. Then, 5 μL of each pre-amplified sample was loaded in duplicate into each sample inlet and 5 μL of each Taqman primer (10x) were loaded into each assay inlet using the Integrated Fluidic Circuit Controller HX machine. Real-time qPCR was performed according to the Biomark GE 96.96 Standard v2 protocol and data were exported using the Fluidigm Real-Time PCR analysis software (v4.1.1). The ΔΔCT method was used to determine the relative gene expression of each tumor group to the 143B cell (Fig. 6a), 143B^143B^ (Fig. 6b), and GBM^100^ group (Fig. 6c, d). The mean expression values of *18SrRNA*, *OAZ1*, and *HPRT1* were used as the internal controls for data normalization. Data were represented as the fold change to the control groups (*n* = 3; mean ± SEM) and statistical significance was determined using one-way ANOVA.

## Additional files

Additional file 1: Read coverage from bisulfite sequencing. (XLSX 11 kb)

Additional file 2: The differences in mtDNA methylation amongst the 143B^143B^, 143B^GBM^ and 143B^NSC^ mitochondrial genomes. (XLSX 59 kb)

Additional file 3: The full potential for DNA methylation at each CpG site in the mitochondrial genomes for each 143B tumor type determined by inducing DNA methylation on their respective long PCR samples. (XLSX 84 kb)

Additional file 4: Primer pairs for long and real-time PCR and pyrosequencing. (XLSX 10 kb)

Additional file 5: Taqman gene expression primers used in the Fluidigm qPCR arrays. (XLSX 9 kb)

## Abbreviations

143B: Osteosarcoma cell line
143B^143B^: Osteosarcoma cell line 143B with mtDNA from 143B cells
143B^GBM^: Osteosarcoma cell line 143B with mtDNA from GBM cells
143B^NSC^: Osteosarcoma cell line 143B with mtDNA from hNSC cells
5Aza: 5-Azacytidine
5hmC: 5-Hydroxymethylcytosine
5mC: 5-Methylcytosine
ATP6: ATP synthase Fo subunit 6
ATP8: ATP Synthase Membrane Subunit 8
bFGF: Basic fibroblast growth factor
CO1: Cytochrome C Oxidase I
CYTB: Cytochrome B
ddC: 2′-3′-dideoxycytidine
DMEM: Dulbecco’s modified Eagle medium
EGF: Epidermal growth factor
ETC: Electron transfer chain
GBM: Glioblastoma multiforme
GBM^0.2^: Tumors formed from cells possessing 0.2% of mtDNA content
GBM^100^: Tumors formed from cells possessing 100% of their mtDNA content
GBM^3^: Tumors formed from cells possessing 3% of mtDNA content
GBM^50^: Tumors formed from cells possessing 50% of their mtDNA content
HIF1α: Hypoxia-inducible factor 1-alpha
hNSC: Human neural stem cells
HSP: Heavy strand promoter
IDH: Isocitrate dehydrogenase
LSP: Light strand promoter
MeDIP: Methylated-DNA-immunoprecipitation
mtDNA: Mitochondrial DNA
ND1: NADH-ubiquinone oxidoreductase chain 1
ND3: NADH dehydrogenase 3
ND4: NADH dehydrogenase subunit 4
ND4L: NADH-ubiquinone oxidoreductase chain 4 L
ND5: NADH:Ubiquinone Oxidoreductase Core Subunit 5
ND6: NADH dehydrogenase 6
O_H_: Origin of heavy strand replication
O_L_: Origin of light strand replication
OXPHOS: Oxidative phosphorylation
POLG: DNA polymerase gamma subunit 1
RNR1: 12S RNA
RNR2: 16S RNA
rRNAs: Ribosomal RNAs
TCA: Citric acid cycle
TET: Ten-eleven translocation methylcytosine dioxygenases
TG: TRNA Glycine
TOP1MT: DNA topoisomerase I mitochondrial
TR: TRNA Arginine
tRNAs: Transfer RNAs
VitC: Vitamin C
